# Cutaneous dirt-adherent disease complicated with Darier’s disease, schizophrenia, and cutis verticis gyrata: A case report

**DOI:** 10.3389/fmed.2022.939107

**Published:** 2022-07-29

**Authors:** Qing Zhu, Shu-Jing Guo, Bin Wang, Lu-Lu Xu, Guo-Qiang Zhang

**Affiliations:** Department of Dermatology, The First Affiliated Hospital of Hebei Medical University, Shijiazhuang, China

**Keywords:** cutaneous dirt-adherent, keratosis follicularis, *ATP2A2* gene, schizophrenia, gyrate cranial skin

## Abstract

The patient was a 25-year-old man presented with cutaneous dirt-adherent disease with a past medical history of schizophrenia. Both the patient and his mother had Darier’s disease, genetic screening revealed that the patient carried a heterozygous frameshift mutation in *ATP2A2* gene, which was inherited from his mother. Cutis verticis gyrata was also found in the patient.

## Introduction

Cutaneous dirt-adherent disease (CDAD) is a rare mental skin disease characterized by skin limitation and persistent adherence to dirt substances. The disease was first reported in 1960 and named in 1964. At present, mental factor is considered to be one of the causes. Darier’s disease (DD) is an inherited skin disease that features blemishes on skin and mucous membranes. DD has also been associated with an increased prevalence of a variety of neuropsychiatric conditions. Here, we report the case of a young man with CDAD and DD and with a past medical history of schizophrenia.

## Case description

The patient was a 25-year-old man presented with recurrent exudation, scab with pruritus and pain in the scalp, face and neck for 9 years, and recurring and aggravating for 2 weeks. The patient developed lesions with exudation on the face and scalp 9 years ago. The lesions have recurred in the past few years, and local skin appeared rough and thickened. Then, 2 weeks ago, the patient was caught in the rain, and lesions on scalp and face increased rapidly with pustules emerging, accompanied by itching, pain, and discomfort. Then, the patient began to develop recurrent high fever (over 40°C) with rigors. He went to a local clinic and was given anti-inflammatory drugs (the specific treatment was unknown) for 4 days, and the symptoms did not improve. Abnormal increase of creatine kinase and transaminase was detected. Then, he was admitted to the intensive care department at the local hospital. In the intensive care department at the local hospital, he was conducted pus bacterial culture and hemoculture, both with negative results. He was diagnosed with sepsis. Then, he received treatment of norvancomycin hydrochloride (0.8 g Q12h), biapenem (0.3 g Q8h) for anti-infective therapy, and Tathion (1.8 g QD) for improving liver function by intravenous infusion for 8 days. Then, fever was reduced, and creatine kinase and transaminase improved significantly. The pustules and pus subsided and evolved into large black scabs. After that he was transferred to the general ward for further treatment for 3 days. Part of the scabs fell off, but there were still a large number of black scabs on the scalp, face, and neck. Then, he was admitted to our department.

Past medical history: approximately 10 months ago, he was in a bad mood due to work and felt that someone wanted to harm him. The patient was diagnosed with schizophrenia in the local hospital and was given medication for more than 5 months. The symptoms did not recur. Later, he had follow-up visit at the hospital and was recommended to stop the medication. The drug had been stopped for more than 2 months up to the admission. Family history: his mother suffered from DD.

**FIGURE 1:**
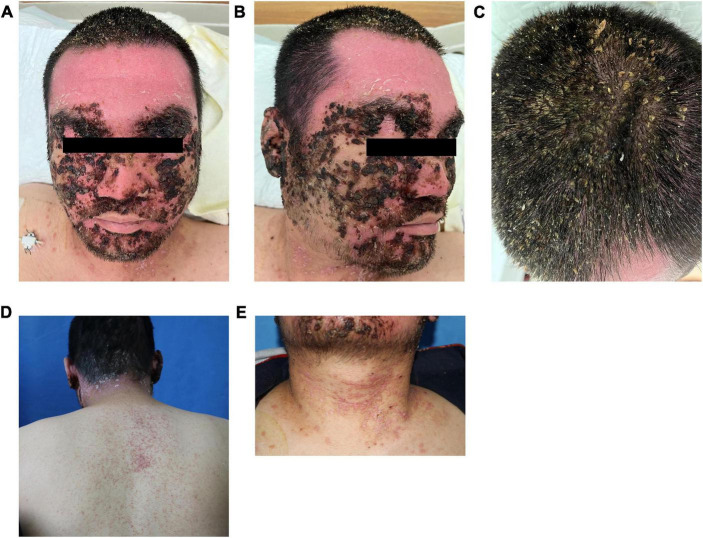
Clinical pictures **(A–E)**. Upon admission, a large number of black crusts were seen on the head and face of the patient, with a large number of yellow greasy crusts attached to the scalp, dense erythema, and isolated follicular papules on the neck and back.

Physical examination showed apathetic facial expressions and silence to the questions. Dermatological examination: yellow to brown serous scab are densely distributed seen on the scalp, the skin of the forehead was dark red, rough, and hypertrophic, dense scales can be observed. Large yellow and black-brown greasy crusts were observed in zygomatic, eyebrow, temporal, buccal, and mandible, which were closely adhered and difficult to peel. Erythema, papules, and greasy crusts were observed in the V zone of neck, fore chest, and back (Figures 1A–E).

**FIGURE 2:**
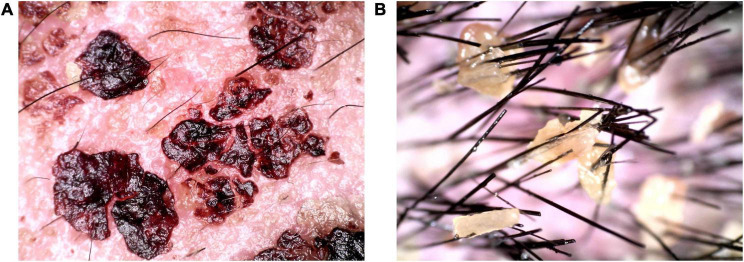
Dermoscopic pictures **(A,B)**. A large amount of dark reddish-brown blood and yellow callus-like substances were observed on the scalp and hair under dermoscopy.

Laboratory examination: blood routine: red blood cell count 3.26 × 10^12^/L (4.3–5.8 × 10^12^/L), hemoglobin 103 g/L (130–175 g/L); rapid c-reactive protein 43.08 mg/L (0–10 mg/L); Biochemistry: γ-glutamyltranspeptidase 161 μ/L (10–60 μ/L), albumin 37.6 g/L (40–55 g/L), alanine aminotransferase 100.8 μ/L (9.0–50.0 μ/L), creatinine 43.1 μmol/L (57–97 μmol/L), lactate dehydrogenase 312.0 μ/L (114–256 μ/L); erythrocyte sedimentation rate: 66 mm/h (0–15 mm/h); urine routine examination, total items of toxoplasmosis, blood coagulation, infectious diseases examination, fungal dextran + bacterial endotoxin + aspergillus, procalcitonin, cytokines, ENA + ANCA full item + antinuclear antibody screening were normal. Pus bacterial culture: there was no bacterial growth. Head CT, chest CT, and abdominal ultrasonography showed no obvious abnormalities. Mycological microscopic examination of skin lesions showed spores positive. Dermoscopy showed dark red and dark reddish-brown crust-like patches of different sizes and irregular shapes can be seen attached to the pink background, as well as a large number of yellow crust-like structures attached to the scalp and hair stem (Figures 2A,B). Histopathological examination of the lesions on the posterior left neck revealed that epidermal hyperkeratosis, irregular hyperplasia, hair follicle horn thrombosis, superficial dermal vascular dilatation, moderate amount of tissue cells, lymphocyte infiltration, no obvious spinous layer lysis, and grain and round body were observed.

The diagnosis was “1. Cutaneous dirt-adherent disease, 2. Schizophrenia, 3. Darier’s disease, 4. Hepatic dysfunction.”

**FIGURE 3:**
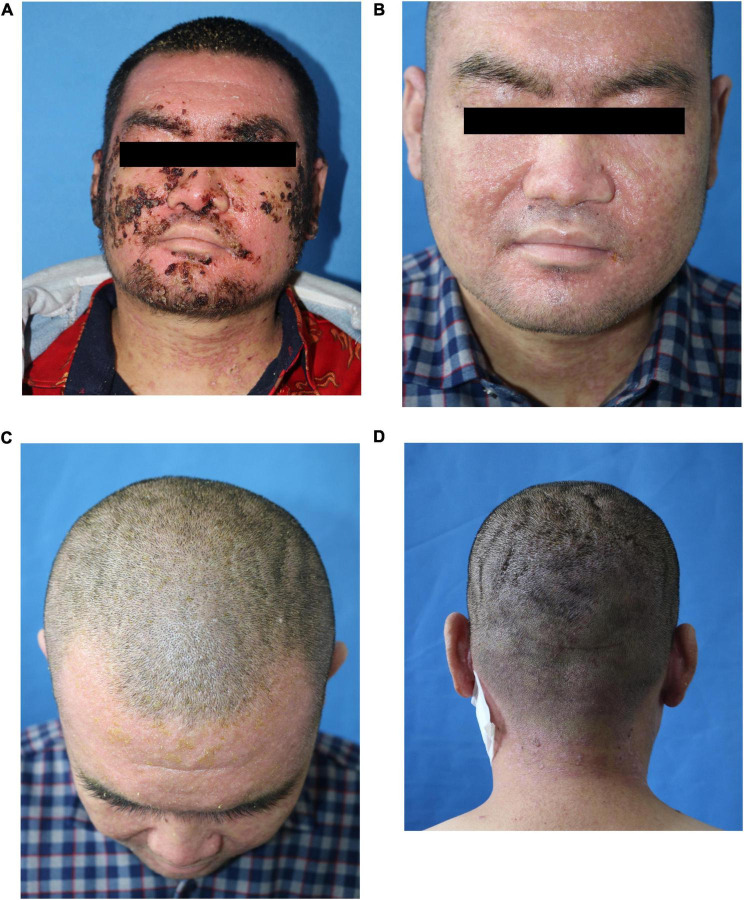
Clinical pictures after treatment. **(A)** Three days after treatment; **(B–D)** the scalp was furrowed before discharge.

We gave the patient compound glycyrrhizin for hepatic dysfunction. Emollient was used on the thick scab on the face and selenium sulfide for the scalp. Approximately 0.1% tacrolimus and ketoconazole cream was given for facial area (Figure 3A). After removing the facial and hair scabs and haircut, we found the convoluted folds and deep furrows of the scalp that mimic cerebral sulci and gyri (Figures 3B–D). A supplementary diagnosis of cutis verticis gyrata (CVG) was made. After treatment, the mental state of the patient was significantly improved compared with that at the time of admission.

**FIGURE 4:**
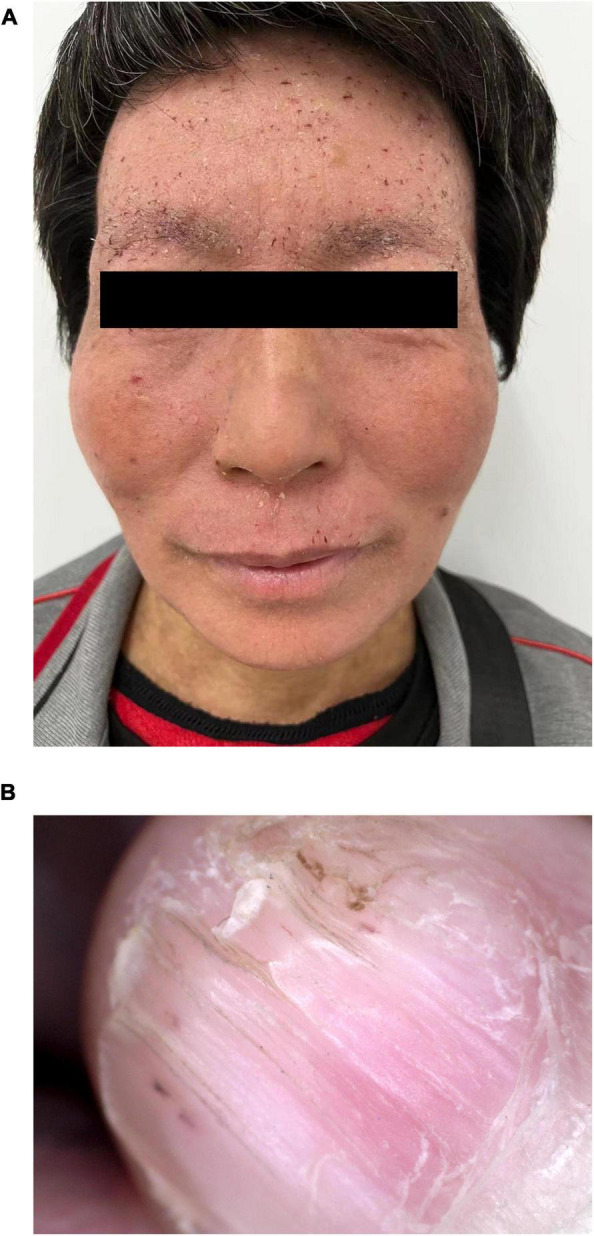
Clinical pictures of the patient’s mother. **(A)** Several isolated keratinized papules were seen densely distributed on the face and neck of the patient’s mother. **(B)** Longitudinal stripes and V-shaped defects were seen on the nails.

The patient’s 50-year-old mother had erythema and papules on her face and neck for 34 years. Facial skin redness appeared 34 years ago, with multiple millet-sized firm papules covered with greasy yellow scales. Around 30 years ago, the facial lesions significantly aggravated. She had been treated with traditional Chinese medicine, and the rash was slightly improved in winter. A pathological biopsy was performed at a local hospital, and she was diagnosed DD. She did not receive any treatment. Dermatological examination showed that rough skin on face and neck, red and light brown erythema, and keratinizing papules could be observed, and dry silver-white scales could be seen on both ears; longitudinal stripes and V-shaped defects were seen on multiple nails (Figures 4A,B).

**FIGURE 5:**
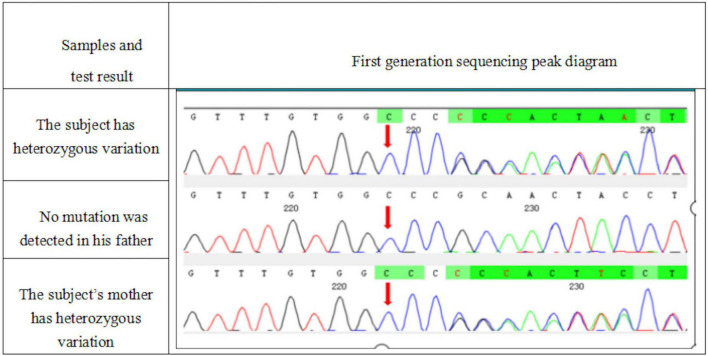
A heterozygous mutation c. 2962dup:p.R988Pfs *15 appeared in the *ATP2A2* gene of the subject. This mutation was a duplication of the 2,962 base of cDNA, resulting in the change of the 988 codon from encoding arginine to encoding proline, and then, a stop codon was generated in advance.

Whole exome sequencing was performed on the patient and his parents, and the results were positive, with genetic variations associated or partially associated with the clinical phenotype of the patients. Gene variation analysis was performed on the whole exome group and adjacent splice region of the family of the subject, it was found that the subject carried a heterozygous code shift variation on *ATP2A2* gene, namely, c. 2962dup: p.R988Pfs *15 (Figure 5), and the results of first-generation sequencing showed that the mutation was reliable and inherited from the mother.

**FIGURE 6:**
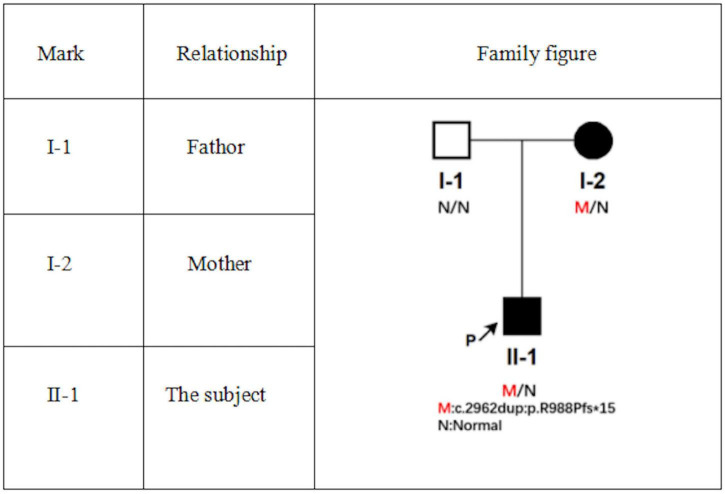
Family diagram of subjects.

At the same time, a heterozygous missense mutation was also found in *KRT86* gene located on *chr12:52695809*, which changed the 109th base of cDNA from T to C, resulting in the change of the 37th codon from cysteine to arginine, which was inherited from the father (Figure 6).

## Discussion

Cutaneous dirt-adherent disease is considered to be a rare disease associated with mental illness, trauma, endocrine disorders, or chronic lack of cleaning, with a predilection for young Japanese and Chinese women (1). In general, it often occurs in the forehead, cheeks, breasts, and other parts of sebaceous glands with strong secretion and also in the palm and scar sites (2). The common clinical features are thick brown verrucous crusts stuck on the surface of the skin, with clear boundaries, and are hard to peel (3). Recently, some literature suggested that the disease is related to Malassezia infection, which may be caused by mental factors that promote the secretion of sebum and provide a better breeding environment for Malassezia. Mental factors may also directly lead to the onset of CDAD. In this case, the patient had a clear history of schizophrenia, the mycological microscopic examination showed spores positive, and the disease may be caused by the two common factors. When the patient’s facial dirt was cleared, the patient’s psychological screening score was reduced, and the anxiety and depression state was improved.

Darier’s disease is a rare autosomal dominant skin disease with epidermal keratosis as the basic change. The disease is autosomal dominant with complete, delayed, and varied penetrance (4). Various studies have confirmed that this disease is caused by mutations in *ATP2A2* gene encoding sarcoplasmic/endoplasmic reticulum calcium-*AT PASE 2* (*SERCA2*) on autosomal *12q23-q24* (4, 5). It may also be related to environment, immune disorders, calcium signal transduction, and apoptosis (6). Skin lesion occurs in sebum overflow sites, such as scalp, forehead, ear, nasolabial groove, neck, shoulder, chest, back, and so on, characteristic rash is solid papules of hair follicles, accompanied by greasy scab, funnel-shaped concave can be observed after removing the scab, and papules can gradually increase and fuse into warty plaques, which can be accompanied by itching and pain. Some may be accompanied by hyperkeratosis and nail damage. Yeshurun et al. divided the cutaneous manifestations of DD into two types of lesions: classical and non-classical. The classical lesions include keratotic papules, acral pits, and acral wart-like lesions, and they are common and characterize the disease, whereas the non-classical lesions including acral keratoderma, leukodermic macules, giant comedones, keloid-like vegetations, and acral hemorrhagic blisters are rare and appear only in patients with DD variants. They also found that all patients (100%) had at least one of the classical lesions and 33% of patients had non-classical lesions of DD (7). According to their classification, we have found that our patient had only classical cutaneous manifestations as described before, but no non-classical lesions. In addition to the typical lesions, different patients may be accompanied by neurological and psychiatric disorders such as epilepsy, schizophrenia, intellectual disability, and so on. Our patient’s mother was a patient with DD. Genetic screening of the family found that the patient had mutations in *ATP2A2* gene, which was inherited from his mother. The family diagram is shown in Figure 6. Referring to the American College of Medical Genetics and Genomics (ACMG) guidelines (8), this variation is a possible pathogenic variation. At present, this mutation has not been reported, which will enrich the mutation database of *ATP2A2* gene and provide help for further research on the relationship between *ATP2A2* mutation and DD. At the same time, missense mutation of *KRT86* gene on chromosome 12 inherited from the father has not been reported in the literature yet. In total, eight kinds of bioinformation prediction software predict that this mutation will cause harmful effects on genes or gene products. It is known that the abnormality of *KRT86* gene can lead to autosomal dominant rosary hair, and the main clinical manifestations of this disease include alopecia, hair dryness, perihair keratosis, hair follicle keratosis, and nail dysplasia. According to ACMG guidelines, this variant is of unknown significance. So we do not know for sure whether this mutation had also caused the disease in our patient.

It has been reported that DD can be combined with psychoneurotic symptoms, and it is found that the risk of schizophrenia in patients with DD is 2.3 times than that of the normal population (9). Some scholars believed that the pathogenesis of DD with neurologic symptoms may be related to *ATP2A2* gene mutation, rather than the patient’s psychological reaction to skin disease (10). Some scholars also believed that DD combined with neurologic symptoms may be related to the pleiotropic expression of *ATP2A2* gene in skin and central nervous system. In addition to keratosis follicularis, SERCA2 dysfunction causes abnormal calcium stability in central nervous system, leading to neurologic symptoms (11). The mutation of Cys318 in *ATP2A2* gene is associated with schizophrenia (12), which still needs further confirmation. Although the patient appeared psychiatric symptoms after DD, the DD history was 9 years, the schizophrenia history was less than 1 year, and there were clear reasons for work difficulties. From this, we speculate that it may not be a psychological response to DD. We should pay attention to the patients’ neuropsychiatric symptoms and evaluate their mental state when seeing patients with DD in the future.

Clinically, the differential diagnosis of DD includes seborrheic dermatitis and Hailey–Hailey disease. Cutaneous manifestation of seborrheic dermatitis is erythema with clearly defined borders arising in seborrheic areas such as scalp, face, fore chest, armpit, groin, etc. and accompanied by sebaceous scales. As for Hailey–Hailey disease, painful rash and blistering in skin folds was characterized. This disease commonly arises during adolescence, with the aggregation of erythema and small blisters at intertriginous areas. Gradually, an ulcerated and macerated surface forms with crusts. The differentiation of Hailey–Hailey disease from DD can be clinically challenging.

As we already know that stress, ultraviolet light exposure, high temperatures, high humidity, excessive sweating, oral contraceptives, pregnancy and delivery, surgery, and mechanical irritation may be the exacerbating factors for DD. In our case, the patient had been exposed to rain, and we think it may be the trigger of the exacerbation. Previous literature reported that a secondary infection of DD could lead to sepsis, and the patients died eventually (13, 14). One study have found that patients with DD had high prevalence of Staphylococcus aureus colonization in lesional skin and nares, with a correlation between disease severity and extent of the colonization (15). When the patient was in the intensive care department of the hospital, he was conducted pus bacterial culture prior to antibiotic treatment, with negative results. He came to our hospital after more than 1 week of anti-infective treatment. In our department, bacterial culture of pus was conducted again before our treatment, and the result was still negative. Regrettably, we did not take samples from the patients’ nares.

Since the patient and his family had not found any furrowed scalp changes before, it was considered to be secondary CVG caused by DD. At present, there is only one case reported abroad (16) with DD combined with gyrus cranial skin. The literature reported a 46-year-old woman with the rare co-occurrence of DD and CVG complicated by hyperprolactinemia and depressive disorder. They attempted to find a single cause behind the different symptoms and performed multiple genetic examinations, and there were no significant findings. Therefore, they supposed that CVG was induced by polyendocrinopathy. In our case, whether the two conditions are related to genes remains to be studied.

To our knowledge, this is the first case of CDAD complicated with familial DD, mainly in the face, neck, and back and accompanied by CVG, and the patient had a history of schizophrenia, which was relatively rare.

## Data availability statement

The datasets presented in this article are not readily available because of ethical/privacy restrictions. Requests to access the datasets should be directed to G-QZ, zlx090702@163.com.

## Ethics statement

Written informed consent was obtained from the individual(s) for the publication of any potentially identifiable images or data included in this article.

## Author contributions

S-JG, BW, and L-LX performed the pathological studies, gene testing, and clinical data collection. QZ was in charge of patient care and drafted this manuscript. G-QZ revised the manuscript critically for important intellectual content. All authors contributed to the article and approved the submitted version.
